# Diagnosis and Personality

**Published:** 1916-05-20

**Authors:** 


### DIAGNOSIS AND PERSONALITY.

The interpretation of symptoms and of facts found on examination of the patient is a large part of the duty of the practitioner of medicine. It is at once the pride of his art and the demand of the public. To reach a diagnosis is universally allowed to be a necessary prelude both to successful treatment and to accurate prognosis. The task is sometimes an easy one; often it is difficult; and sometimes it is impossible. A broken limb is generally within the compass of a first-aid student, but pains and sensations and discomforts offer a larger field of inquiry. And there are complexities and apparent contradictions which puzzle even the elect. Such happenings form the opportunities for those differences of opinion of which doctors are proverbially accused. To the man in rude health these differences are a proof of the stupidity and incapacity of the profession. In his own affairs he is confident to the point of cocksureness. Were the doctors equal to him in knowledge and judgment, they also would be without hesitation. The verdict is a summary one, and probability opposes it. On the face of things, it is unlikely that members of the medical profession generally are more stupid than their neighbours. Hence the true explanation of their hesitations and uncertainties is, not that they are less capable or less industrious than their more confident critics but that the problems which confront them are more difficult. Ingenuity and skill are constantly endeavouring to interpret these problems, and the resources of many arts are pressed into this service. That numerous advances and gains can be chronicled, none will dispute, but there remains one difficulty which cannot be fully measured, and, indeed, is never likely to be fully measured. It is the quality impressed upon the case by the personality of the patient. Were human beings as monotonous as bricks, or as uniform as test-tubes, medical practice, though much less entertaining, would certainly be more definite. But to the end of the chapter Brown will always be himself, and never Jones or Robinson. Therefore we take our material as we find it, and, with all its peculiarities, we would not have it otherwise. Though we cannot be as exact as the plumber or as precise as the lawyer we cultivate an approximate estimate of human nature and we recognise how easily we may be deceived.

After all, there is much in modern medicine which is exact enough to satisfy the most ardent devotee of weight and measure. Instruments and methods of precision multiply day by day, and records can be inscribed in characters beyond challenge. Diagnosis has thus become both more penetrating and more graphic, and the gain is great both to doctor and to patient. The movement, without doubt, will extend, and none but a very rash man would pretend to fix its limits. Even at the best, however, the personality of the patient will remain as a more or less ill-defined factor in the problem, and in the judgment of it there will be room for hesitations and uncertainties. Here help will come, not from machines and tests but from native shrewdness and well-used experience.

